# Structural changes on a molecular basis of canola meal by conditioning temperature and time during pelleting process in relation to physiochemical (energy and protein) properties relevant to ruminants

**DOI:** 10.1371/journal.pone.0170173

**Published:** 2017-02-16

**Authors:** Xuewei Huang, Huihua Zhang, Peiqiang Yu

**Affiliations:** 1 Department of Animal and Poultry Science, College of Agriculture and Bioresources, University of Saskatchewan, Saskatoon, Saskatchewan, Canada; 2 College of Life Science and Engineering, Foshan University, Guangdong, China; Laval University, CANADA

## Abstract

The objectives of this study were: (1) To investigate the effects of conditioning temperature (70, 80, 90°C), time (30, 60 sec), and interaction (temperature × time) during the pelleting process on internal protein molecular structure changes of the co-products; (2) To identify differences in protein molecular structures among pellets that were processed under different conditions, and between unprocessed mash and pellets; 3) To quantify protein molecular structure changes in relation to predicted energy and protein utilization in dairy cows. The final goal of this program was to show how processing conditions changed internal feed structure on a molecular basis and how molecular structure changes induced by feed processing affected feed milk value in dairy cows. The hypothesis in this study was that processing-induced protein inherent structure changes affected energy and protein availability in dairy cattle and the sensitivity and response of protein internal structure to the different pelleting process conditions could be detected by advanced molecular spectroscopy. The protein molecular structures, amides I and II, amide I to II ratios, α-helix structure, β-sheet structure, and α to β structure ratios, were determined using the advanced vibrational molecular spectroscopy (ATR-FT/IR). The energy values were determined using NRC2001 summary approach in terms of total digestible nutrients, metabolizable and net energy for lactation. The protein and carbohydrate subfactions that are related to rumen degradation characteristics and rumen undegraded protein supply were determined using updated CNCPS system. The experiment design was a RCBD and the treatment design was a 3x2 factorial design. The results showed that pelleting induced changes in protein molecular structure. The sensitivity and response of protein inherent structure to the pelleting depended on the conditioning temperature and time. The protein molecular structure changes were correlated (*P* < 0.05) with energy values and protein subfractions of the pelleted co-product. The results indicated that the protein internal molecular structure had significant roles in determining energy and protein nutritive values in dairy cows. Multi-regression study with model variables selection showed that the energy and protein profiles in pelleted co-products could be predicted with the protein molecular structure profiles. This approach provides us a relatively new way to estimate protein value in dairy cows based on internal protein molecular structure profile.

## Introduction

Bio-energy production (e.g. bio-ethanol, bio-fuel, bio-oil) resulted in millions of tonnes of different types of co-products such as carinata meal, canola meal and distiller's dried grains with solubles [1–7].

It is important to reveal and understand how processing affects inherent structures of these co-products in relation to nutrient utilization and availability in dairy cattle for accurate diet formulation. However, conventional chemical analysis can only reveal how the processing affects total chemical composition of co-products, but it cannot reveal inherent structural changes induced by the processing. Conventional feed evaluation by using wet chemistry with harsh chemical reagents always destroys inherent molecular structure of feeds [8].

Pelleting is a common feed processing method. To fully understand the effect of pelleting under different conditions on nutrient digestion of co-products from bio-oil/bio-fuel processing, we rely on not only information of total chemical composition changes during the processing but also information of how inherent structure changes induced by the processing on a molecular basis [9–12].

Advanced vibrational molecular spectroscopy, which is able to explain and map feed structures and quantify protein inherent structure, can be applied in feed structure analysis [13–15]. ATR-FT/IR, which is based on internal reflectance method, is one kind of advanced FT/IR spectroscopy that has been used recently in feed analysis with different feed types [3, 6, 16–28].

Recently we reported the effects of canola meal pellet conditioning temperature and time on ruminal and intestinal digestion, hourly effective degradation ratio, and potential nitrogen to energy synchronization in dairy cows [11]. However, no systematical study was conducted to determine how the pelleting at different conditioning temperature and time induce the changes in protein intrinsic molecular structures in the co-products and how these molecular structure changes affect energy and protein utilization in dairy cows. The objectives of this study were 1) to detect protein molecular structure of a co-product (canola meal) processed by pelleting under different conditioning temperature and time; 2) to identify differences in protein molecular structures of the co-product among pellets under different processing conditions, and between unprocessed mash and pellets; 3) to quantify protein inherent structure changes in relation to protein chemical composition, CNCPS protein fractions and energy content. The hypothesis in this study was that the processing-induced inherent molecular structure changes had significant relationship to nutrient profiles in dairy cattle and the molecular structure spectral parameters could be used as accurate predictors to nutrient availability in dairy cattle.

## Materials and methods

### Sample preparation, pelleting process, and pellet durability test

The pelleting conditions and sample preparation were reported previously [11] as follow: “The co-product from bio-oil processing—canola meal were used in this study to investigate on the effect of conditioning temperature, time and their interaction of pelleting process on internal protein structure changes on a molecular level in relation to nutrient nutritional value in dairy cattle. Two representative sources of the co-product were obtained from Federated Cooperatives Limited (Saskatoon, SK, Canada) with different manufacturing dates. A California laboratory pellet mill (NH-397202, California Pellet Mill Co., Crawfordsville, IN, USA) with a capacity of 20 kg/h was used to produce canola meal pellets. The conditioning temperature (70, 80, 90°C) was set with heating pads wrapped around the conditioning chamber, and maintained with an Omega CN350 controller (OMEGA Engineering Inc., Stamford, CT, USA). The temperature of the conditioned mash was measured at the outlet of the conditioner continuously by an OMEGA HH-25TC thermometer. Conditioning time was determined by changing the flow rate at which the canola meal entered the conditioner. The conditioning time was determined by placing colored materials into the conditioner, then measuring time until the materials exited the conditioner. At dial settings of “24” and “30” on the pelleting machine, the production rates were 250 and 814 g/min, respectively; and related transition time of materials in the conditioner were 60 and 30 sec, respectively. Steam was injected at 36 psi during the conditioning stage. A pellet die with a hole diameter of 6.4 mm and a thickness of 44 mm was used. Pellets were cooled before the pellet durability test by exposing pellets to the air for 24 hours. This resulted in 12 treatment samples (3 temperatures × 2 time × 2 different batches). The pellet durability was presented as a Pellet Durability Index (PDI), which was measured using the “Pfost” method and expressed on a percentage basis [29]. Around 500 g of sieved pellets were put in a drum with specified dimensions (30.5 × 30.5 × 12.7 cm) and tumbled for 10 min at 50 rpm. After tumbling, the pellets were screened with a sieve in grid size to determine the pellets which could not pass through sieve. PDI was calculated as [29]*”*. The physiochemical property data were used for relationship study between pelleting induced molecular structural changes in relation to nutritive values in ruminant animal -dairy cattle.

### Pelleting induced changes in chemical profiles of co-product from bio-oil processing

Detailed chemical analyses were reported previously [11, 12] as follow: “For chemical analyses, all samples were ground through a 1 mm screen using a Retsch ZM 200 Rotor mill (Rose Scientific Ltd., Edmonton, AB, Canada). Dry matter (DM), ash, ether extract (EE), and crude protein (CP) were analyzed according to AOAC official methods 930.15, 942.05, 954.02, and 984.13, respectively. The methods described in Van Soest et al. [30] combined with an ANKOM A200 filter bag technique (ANKOM Technology Corp., Fairport, NY, USA) were used to determine acid detergent fiber (ADF), neutral detergent fiber (NDF), and acid detergent lignin (ADL) in the samples. Adjusted NDF was determined when sodium sulfite was added together with heat-stable amylase before neutral detergent extraction. The methods in Licitra et al [31] were applied to analyze acid detergent insoluble crude protein (ADICP) and neutral detergent insoluble crude protein (NDICP). All samples were incubated with a bicarbonate-phosphate buffer, then filtered through the Whatman #54 filter papers to determine the total soluble crude protein (SCP) content [32]. The method used to determine non-protein nitrogen (NPN) was in accordance with Licitra et al [31], in which tungstic acid is used to precipitate the true protein fraction and then the difference between total crude protein and precipitated crude protein is considered the NPN.” The detailed chemical profiles and PDI changes induced by different conditioning temperatures and time of pelleting process were reported previously [11, 12]. These data were used for relationship study between pelleting induced molecular structural changes in relation to predicted protein and energy values in dairy cattle.

### Pelleting induced changes in energy values of co-product from bio-oil processing

Detailed methods were reported previously [11, 12] as follow: “summative approaches from the National Research Council [33] dairy were used to estimate values of the total digestible non-fiber carbohydrates (tdNFC), the total digestible crude protein (tdCP), the total digestible neutral detergent fiber (tdNDF), the total digestible fatty acid (tdFA), the total digestible nutrients at 1× maintenance (TDN_1x_), the total digestible nutrients at 3× maintenance (TDN_3x_), the digestible energy (DE_1x_), the digestible energy at the production level of 3×maintenance (DE_p3x_), the metabolizable energy at the production level of 3× maintenance (ME_p3x_), and the net energy at the production level of 3× maintenance (NE_Lp3x_). The methods to determine the metabolizable energy (ME), the net energy for maintenance (NE_m_) and the net energy for gain (NE_g_) were in accordance with NRC [33].” The energy value changes induced by different conditioning temperatures and time were reported previously [11, 12]. These data were used for relationship study between pelleting induced molecular structural changes in relation to predicted protein and energy values in dairy cattle.

### Pelleting induced changes in protein and carbohydrate fractions of co-product from bio-oil processing

The Cornell Net Carbohydrate and Protein System (CNCPS) v.6.1 was applied to partition the CP and carbohydrate (CHO) fractions [34, 35]. Here is a brief procedure that reported previously [11, 12]: “In the CNCPS v6.1, protein fractions are profiled based on their ruminal degradation characteristics: ammonia (PA1), non-ammonia soluble true protein (PA2), moderately degradable true protein (PB1), slowly degradable true protein (PB2), and completely undegradable CP (PC) [35]. The PA1 degrades at 200%/h. The PA2 degrades at 10 to 40%/h. The PB1 contains neutral detergent soluble protein, and degrades at 3 to 20%/h. The PB2 contains proteins that bind in fiber and slowly degrades at 4 to 9%/h in forages. The CHO fraction scheme is expanded into eight fractions: volatile fatty acids (VFAs) (CA1), lactate (CA2), organic acids (CA3), sugars (CA4), starch (CB1), soluble fibers (CB2), slowly degradable and available NDF (CB3) and lignin (CC). The CA2 degrades at 7%/h, CA3 degrades at 5%/h, and CA4 degrades at 40 to 60%/h. The CB1 is starch and degrades at 20 to 40%/h, and CB2 degrades at 20 to 40%/h. The CB3 has a degradation rate of 4 to 9%/h. Fractional degradation and passage rates were obtained from the CNCPS feed library and literature [34, 35]. In canola meal, CA1, CA2, and CA3 are not presented, and only a small amount of CB1 (5%) was found (*36*). CHO fractions focused on in this study were CA4, CB2, CB3, and CC [6, 7, 36]. Fractions of rumen degradable and undegradable protein and carbohydrate were calculated as well. Models in CNCPS v6.1 are more sensitive to feeds with high levels of protein and carbohydrates [34, 35]. In soluble pools, in order to get a more appropriate reflection of cattle biology, re-assignation of the liquid passage rate equations to CHO CA, PA, and PB has been made [34]. The resultant higher predicted outflow of soluble components would reduce estimated microbial yield and ammonia production [34]” The CNCPS protein subfraction changes induced by different conditioning temperatures and time were reported previously [11, 12]. These data were used for relationship study between pelleting induced molecular structural changes in relation to predicted protein and energy values in dairy cattle.

### Pelleting induced changes in molecular spectral profile of co-product from bio-oil processing with ATR-FT/IR molecular spectroscopy

Pellets produced under different conditioning temperatures (70, 80, 90°C) and time (30, 60s) and unprocessed mash were used in this study. All samples were ground through a 1 mm screen using a Restch ZM 200 roter mills (Rose Scientific Ltd., Edmonton, AB, Canada). The ATR-FT/IR analysis was performed at molecular spectroscopy lab, Department of Animal and Poultry Science, College of Agricultural and Bioresources, University of Saskatchewan, Saskatoon, SK, Canada using a JASCO FT/IR-4200 (JASCO Corporation, Tokyo, Japan) equipped with a ceramic IR light source and a deuterated L-alanine doped triglycine sulfate detector that contains a MI Racle^™^ attenuated total reflectance (ATR) accessory module and outfitted with a ZnSe crystal and pressure clamp (PIKE Technologies, Madison, WI, USA). Spectra were collected at the mid-IR infrared region from ca. 4000–700 cm^-1^ with a spectral resolution of 4 cm^-1^ with 128 scans co-added. To minimize infrared absorption by CO_2_ and water vapour in the ambient air, background spectra were collected with 256 scans. JASCO Spectra manager II software was used as a tool to collect spectra data. Spectral data of each sample was collected five times. OMNIC 7.3 (Thermo-Nicolet, Madison, WI, USA) software was used to identify and collect spectral information of functional groups associated with macromoleculars-protein, cellulosic, and carbohydrate. Absorption peak parameters such as center region, baseline region, height and area were determined according to previous researches [4, 6, 7, 18–20, 25–27, 37, 38].

### Univariate spectral analysis on effect of pelleting on protein molecular structure

The spectral band information of protein related chemical functional groups can be detected on the spectrum according to published literatures [6, 23, 25–27, 37]. For identification of protein secondary structure, the baseline was set ca. 1718–1481 cm^-1^. Peptide bonds, which include C = O, C-N and N-H, play a unique role in protein structure. Amide I consists of 80% C = O stretching vibrations and 20% C-N stretching vibrations, while amide II consists of 60% N-H bending vibrations and 40% stretching vibrations [9, 13, 39–41].

**Fig 1:**
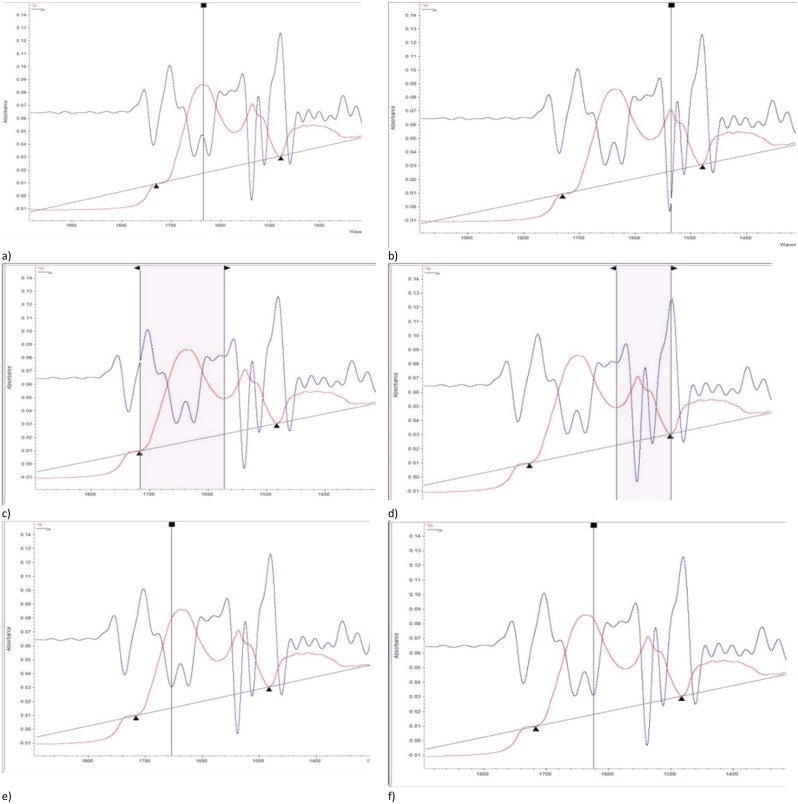
Typical FTIR spectrum for co-products from bio-oil processing at the regions of protein (ca. 1718–1481 cm^-1^). a) amide I height; b) amide II height; c) amide I area; d) amide II area; e) α-helix height; f) β-sheet height.

Band absorption selection of amide I, amide II, α-helix and β-sheet was according to published literature [13, 23, 25, 39]. Therefore, for amide I, peak center ranged ca. 1644–1625 cm^-1^ and area ranged ca. 1718–1567 cm^-1^. For protein amide II, peak center ranged ca. 1538–1535 cm^-1^, and area ranged ca. 1567–1481 cm^-1^. Amide I and amide II are two major bands associated with protein secondary structure [9, 13]. However, amide I is often used in the research of protein structure rather than amide II, this is because amide II comes from complex vibrations that include multiple functional groups and has lower accuracy in predicting protein structure [39]. Amide I can be further divided into several multi-components peak by using second derivative function in OMNIC 7.3, then α-helix and β-sheet were observed among these multi-components. α-helix peak center was located at ca. 1660–1650 cm^-1^, and β-sheet peak center was located at ca. 1627–1618 cm^-1^. These spectral bands related to protein macromolecular were showed in Fig 1.

### Multivariate molecular spectral analysis on intrinsic structure changes

Two different multivariate methods were employed in the current study to perform multivariate spectral analysis using Statistical 8.0 (StatSoft Inc., Tulsa, OK, USA). Agglomerative hierarchical cluster analysis (CLA), which uses Ward’s Algorithm method without parameterization for clusting, presents results as dendrograms [42–44]. Principal Component Analysis (PCA), which is the other multivariate analysis method, transforms all interrelated variances into new uncorrelated variances called principles components (PCs) [42–44]. The result of PCA is presented as a scatter plot using two main PCs, which took more than 95% of variance, in form of PC1 vs. PC2. The detailed procedures of these two methods were reviewed in a series of papers [42–44]. Functional groups of protein (ca. 1718–1567 cm^-1^), cellulosic compounds (ca. 1302–1186 cm^-1^), structural CHO (ca. 1488–1186 cm^-1^), and total CHO (ca. 1186–881 cm^-1^) were analyzed in multivariate molecular spectral analysis.

### Statistical analysis

For univariate molecular analysis, the experiments were designed using a randomized complete block design (RCBD) with a 3 × 2 factorial treatment arrangement. Statistical analyses were performed through MIXED procedure of SAS 9.3 (SAS Institute, Inc., Cary, NC, USA) using the following model:

$$y_{ijkrt}=\mu \;+\;\alpha_{i}+\;\beta_{j}\;+\;\gamma_{k}+\;\rho_{\gamma }\;+\;e_{eijkrt}$$

In which, *y*_ijkrt_ was an observation of the dependent variable *ijkrt*; *μ* was the population mean for variable; *α*_*i*_ was the effect of conditioning temperature; *β*_*j*_ was the effect of conditioning time; *γ*_*k*_ is the interaction of conditioning time and temperature; *ρ*_*r*_ was the effect of batches as a random effect; *e*_*ijkrt*_ was the random error associated with observation *ijkrt*. The three assumptions for RCBD model (residue data normality, common variance and random effect normality) were checked and tested using Proc Univariate with Normal and Plot options with Shapiro-Wilk test. The common variance was checked using residue analysis by checking the residue data against treatment ID.

In addition, polynomial contrasts (linear and quadratic) of conditioning temperature were analyzed when the effect of conditioning temperature was significant. The contrast was performed to detect the difference between the unprocessed and pellets. The significant level for all statistical analyses was declared at *P* < 0.05 and trended at *P* < 0.10. Treatment means were compared using Tukey-Karmer method.

The correlation between protein molecular spectral profiles (protein functional groups) and protein and energy values in dairy cows were analyzed using the PROC CORR procedure in SAS 9.3 (SAS Institute, Inc., Cary, NC, USA). Rank correlation with SPEARMAN option was employed in the correlation study. The normality test of the data for correlation study was carried out with UNIVARIATE option.

Multi-regression with PROC REG procedure with model variables in SAS 9.3 was used to select the most important parameters. The model used was showed as follows:

$$\text{y }=\text{ a}+b_{1}\times x_{1}+b_{2}\times x_{2}+\ldots +b_{n}\times x_{n}$$

The STEPWISE option with “SLENTRY = 0.05, SLSTAY = 0.05” was used to determine selection criteria. Hence, variables left in the model after selection were significant at *P* < 0.05. Then, UNIVARIATE and PLOT options were used to do residual analysis. VIF option in SAS 9.3 was used to detect Collinearity, which represented interrelationship among the protein and carbohydrate structure parameters in our study.

## Results and discussion

### Determining protein molecular spectral characteristics of co-product at amide I and amide II region using univariate molecular analysis

Effects of pelleting under different conditions on protein spectral profiles were briefly reported previously [11]. Briefly, in protein amide spectral region (ca. 1481–1718 cm^-1^), the amide I area was not significantly different among the samples, indicating the pelleting process in our study did not affect amide I area of the co-product. Conditioning time and temperature showed significant (*P* < 0.05) effects on amide II area, but the interaction of time and temperature was not significant. Samples conditioned at 70°C had highest amide II area among the samples conditioned at different temperatures. In addition, samples conditioned for 30s were lower in amide II area than those conditioned for 60s (1.92 vs. 2.10; *P* < 0.05). The peak height of amide II was also significantly affected by conditioning temperature (*P* < 0.01) and time (*P* < 0.01), though their interaction was not significant (*P* > 0.05). The samples conditioned at 70°C had highest peak height of amide II among samples conditioned at different temperatures. Moreover, the samples conditioned for 60s had higher peak height of amide II than those conditioned for 30s (0.041 vs. 0.036; *P* < 0.01). It was observed that the ratio of amide I height to amide II height and the ratio of amide I area and amide II area among the pellets area were significantly affected by conditioning temperature and time, but the interaction of conditioning temperature and time was not significant. The samples conditioned at 90°C had highest ratio of amide I to amide II height (2.54, *P* < 0.05) and highest ratio of amide I to amide II area (1.45, *P* < 0.05) compared with those conditioned at 70°C and 80°C. The increase in conditioning time resulted in reducing the ratio of amide I to amide II height (1.56 vs. 1.41; *P* < 0.001) and ratio of amide I to amide II area (2.48 vs. 2.35; *P* < 0.01) in the current study. Compared with the unprocessed co-product, pellets exhibited significant differences in these two ratios (*P* < 0.05). This was supported by previous literature, which reported that the ratio of amide I to amide II can be significantly affected by feed materials, heat processing and gene transformation [40, 45, 46]. In our study, different conditioning temperature and time of pelleting process resulted in differences in spectral profiles of co-product (canola meal) in terms of height and area ratios of amide I to amide II. Theodoridou and Yu [7] observed that canola meal had lower area ratio of amide I to amide II than canola presscake, indicating that the ratio of amide I to amide II area was reduced after desolventizer toasting during canola processing. However, Samadi et al. [47] reported that no difference in the ratio of amide I to amide II area was observed when canola seeds were autoclaved (121°C; 1 h) and roasted (120°C; 1 h) were compared with unprocessed canola seeds.

### Determining protein α-helix and β-sheet characteristics of co-product

With respect to protein α-helix and β-sheet height, there was no significant difference among all samples. Though there was no significant difference among pellets in ratio of α-helix and β-sheet, the unprocessed co-product was significantly different from pellets in the ratio of α-helix and β-sheet *(P* < 0.05). The unprocessed co-product was lower in the ratio of protein α-helix and β-sheet than pellets. Doiron et al. [40] indicated that nutritive values of feeds in terms of truly absorbed protein supply (MP or DVE value) can be highly altered by the changes of the ratio of α-helix to β-sheet. Yu [43] indicated that lower protein value can be caused by increased content of β-sheet structure that can reduce accessibility of protein to gastrointestinal enzymes. Yu et al. [14] observed that the feather, which had a high protein content of 84%, had very low protein digestibility of 5%. This was partially because very high β-sheet percentage of around 88% was observed in that study. Theodoridou and Yu [7] reported that yellow-seeded canola meal had higher β-sheet value than brown-seeded canola meal. Thus, yellow-seeded canola meal had lower rumen degradability but higher intestinal digestibility of CP than brown-seeded canola meal. Theodoridou and Yu [7] also found that canola meal had lower α-helix height and β-sheet height than canola presscake after dissolvent-toasting at around 95 to 115°C. Yu [43] indicated that increase in β-sheet height value after heat treatment was likely due to protein aggregation after heating to form intermolecular β-sheet structures. However, equivocal results related to effects of processing conditions on protein inherent structure were observed in a previous study due to different heat processing methods [7]. In the research conducted by Yu [43], reduced α-helix and increased β-sheet were observed after golden flaxseeds were roasted. In addition, the ratio of α-helix to β-sheet was decreased from 1.28 to 0.73 in that study. In the study by Doiron et al. [40], increased α-helix to β-sheet ratio was observed in the endosperm area of autoclaved flaxseed (120°C; 20, 40 and 60 min) using synchrotron based FTIR method (SFTIRM). In the study of Samadi and Yu [17], both autoclaving (120°C; 1 h) and roasting (120°C; 1 h) reduced α-helix and β-sheet heights of soybean seeds. In addition, the ratio of α-helix to β-sheet in soybean seed was reduced by autoclaving at 120°C for 1 h. Samadi et al. [47] observed there was no difference in heights of α-helix and β-sheet among raw, autoclaved and roasted canola seeds. However, ratio of α-helix to β-sheet was decreased by autoclaving at 121°C for 1 h, but increased by roasting at 120°C for 1 h. In a recent study done by Peng et al. [27], the ratio of α-helix to β-sheet in camelina seeds was reduced by autoclaving at 120°C for 60 min. However, ratio of α-helix to β-sheet was not changed by roasting at 120°C for 60 min. In our study, lower ratio of α-helix to β-sheet was observed in the unprocessed co-product, which is consistent with the higher rumen degradation and lower intestinal digestion of protein found in pellet samples during the *in situ* and the *in vitro* experiments. Theodoridou and Yu [7] indicated the reason for the changes of α-helix to β-sheet ratio after heat processing was likely due to the denaturation of protein during the heating processing.

### Determining protein molecular spectral characteristics of co-product at amide I and amide II regions using multivariate molecular spectral analysis

The location of function groups and band patterns determined the accuracy of univariate molecular analysis [42, 43, 48, 49]. Two different multivariate analysis methods were applied to reveal differences in molecular structures between the unprocessed co-product and pellets produced under different conditions. One was agglomerative hierarchical cluster analysis (CLA), which uses Ward’s algorithm method without any prior parameterization of the spectral data and displays result as dendrograms [42, 43, 49]. The other one was principal component analysis (PCA), which is based on the statistical reduction method and transforms original data set to new data sets that have uncorrelated variances and retain information of original variances as much as possible at the same time. Scatter plots between selected PCs were used to present the result of PCA [42, 43, 39].

**Fig 2:**
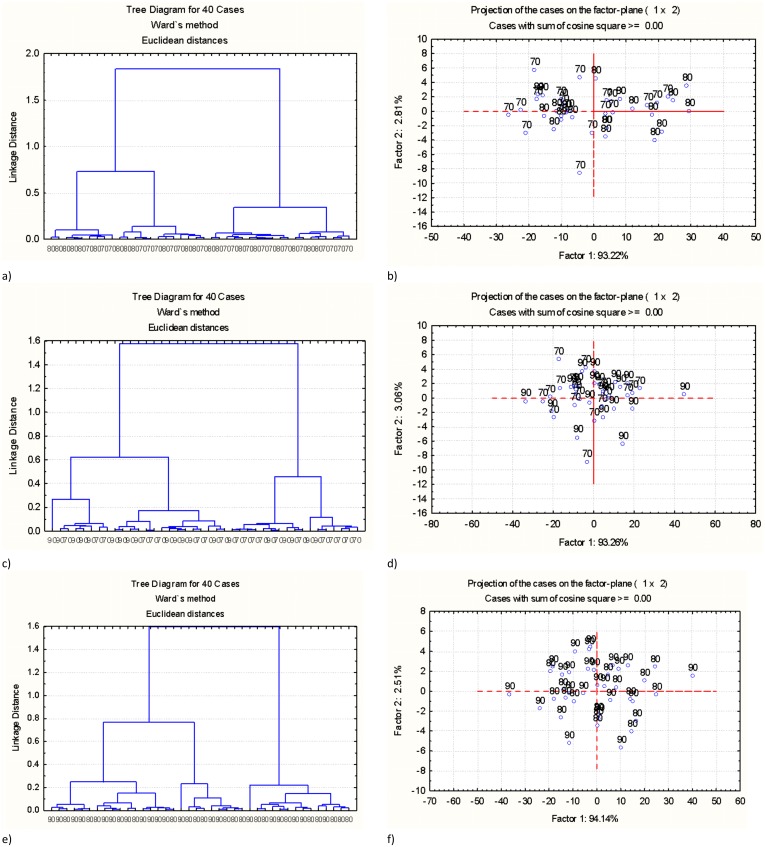
Multivariate molecular spectral analyses of protein fingerprint region at ca. 1718–1481 cm^-1^. Comparison of samples processed under different temperatures using cluster analysis (cluster method: Ward's algorithm and distance method: Euclidean) and principal component analysis [Scatter plots of the 1st principal components (PC1) vs. the 2nd principal components (PC2)]: (a) CLA: samples conditioned at 70°C (70) vs. samples conditioned at 80°C (80); (b) PCA: samples conditioned at 70°C (70) vs. samples conditioned at 80°C (80); (c) CLA: samples conditioned at 70°C (70) vs. samples conditioned at 90°C (90); (d) PCA: samples conditioned at 70°C (70) vs. samples conditioned at 90°C (90); (e) CLA: samples conditioned at 80°C (80) vs. samples conditioned at 90°C (90); (f) PCA: samples conditioned at 80°C (80) vs. samples conditioned at 90°C (90).

**Fig 3:**
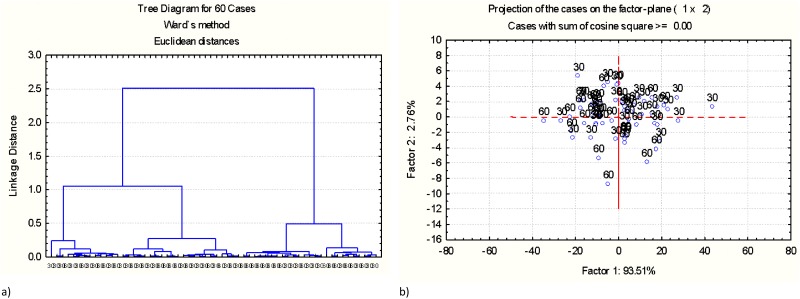
Multivariate molecular spectral analyses of protein fingerprint region at ca. 1718–1481 cm^-1^. Comparison of samples processed under different time using cluster analysis (Cluster method: Ward's algorithm; Distance method: Euclidean) and principal component analysis [Scatter plots of the 1st principal components (PC1) vs. the 2nd principal components (PC2)]: (a) CLA: samples conditioned for 30s (30) vs. samples conditioned for 60s (60); (b) PCA: samples conditioned for 30s (30) vs. samples conditioned for 60s (6030).

Multivariate molecular spectral analyses of the protein spectral region (ca. 1718–1481 cm^-1^) on a molecular basis among the samples are showed in Figs 2 and 3. Fig 2 showed comparisons between samples conditioned at different temperatures in protein spectral region. Results from CLA indicated that no separate class could be distinguished. No separate class could be distinguished between the samples conditioned at 70°C and 80°C [Fig 2(a)]. Similarly, in Fig 2(c), the samples conditioned at 70°C cannot be distinguished from those conditioned at 90°C due to mixed cluster dendrograms. In Fig 2(e), it was unable to fully separate the samples conditioned at 80°C from those conditioned at 90°C. In Fig 3(a), no separate class can be distinguished between the samples conditioned for 30s and those conditioned for 60s. Therefore, CLA showed the similarity of protein structure among pelleted samples processed under different conditions. Pelleted samples processed under different conditions were not completely different in protein structure. This was supported by PCA analysis. In Figs 2(b), 2(d), 2(f) and 3(b), ellipses were overlapped and cannot be fully separated, thereby, again, indicating that samples conditioned under different conditions had a similar relationship in the make-up of protein molecular structure.

**Fig 4:**
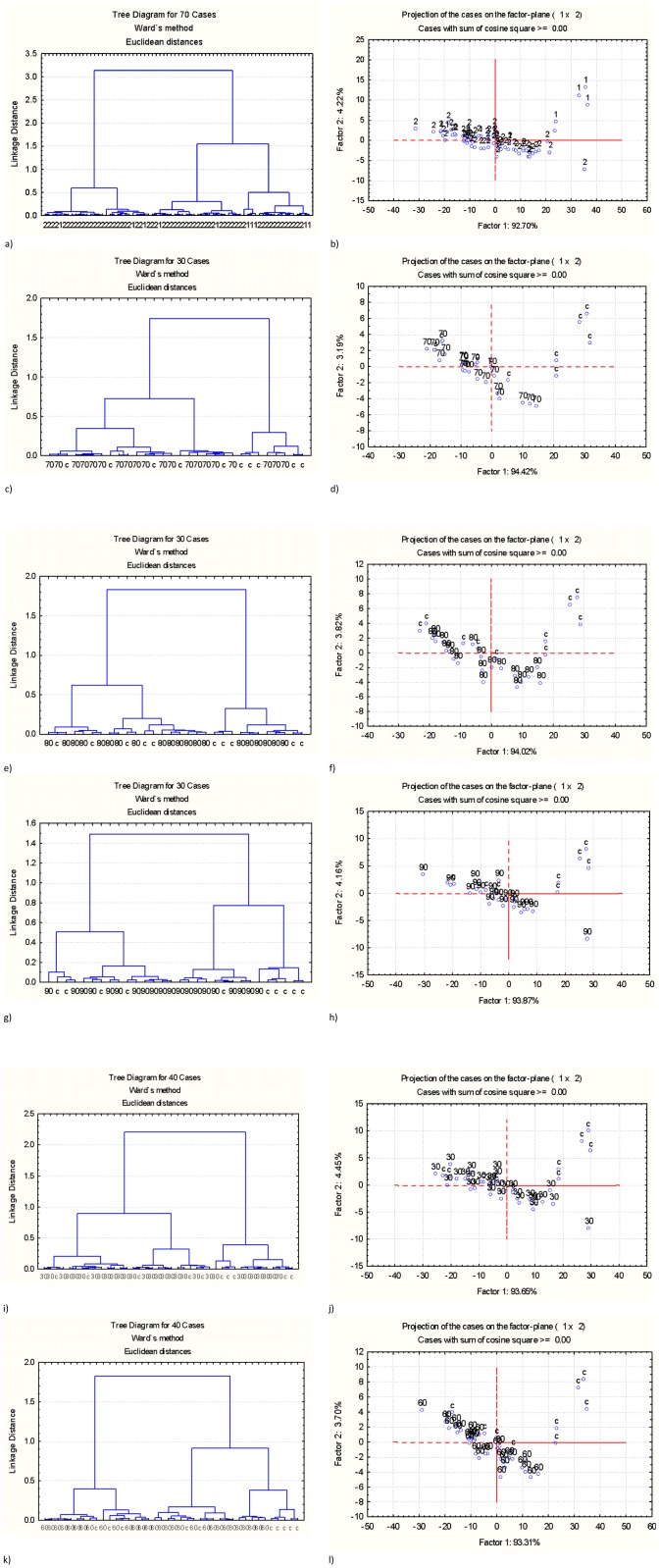
Multivariate molecular spectral analyses of protein fingerprint region at ca. 1718–1481 cm^-1^. Comparison of the unprocessed mash to samples conditioned under different conditions using cluster analysis (Cluster method: Ward's algorithm; (2) Distance method: Euclidean) and principal component analysis [Scatter plots of the 1st principal components (PC1) vs. the 2nd principal components (PC2)]: (a) CLA: unprocessed mash (1) vs. pellets (2); (b) PCA: unprocessed mash (1) vs. pellets (2); (c) CLA: unprocessed mash (c) vs. samples conditioned at 70°C; (d)PCA: unprocessed mash (c) vs. samples conditioned at 70°C; (e) CLA: unprocessed mash (c) vs. samples conditioned at 80°C; (f) PCA: unprocessed mash (c) vs. samples conditioned at 80°C; (g) CLA: unprocessed mash (c) vs. samples conditioned at 90; (h) PCA: unprocessed mash (c) vs. samples conditioned at 90°C; (i) CLA: unprocessed mash (c) vs. samples conditioned for 30s; (j) PCA: unprocessed mash (c) vs. samples conditioned for 30s; (k) CLA: unprocessed mash (c) vs. samples conditioned for 60s; (l) PCA: unprocessed mash (c) vs. samples conditioned for 60s.

With regard to the difference between the unprocessed co-product (c) and pellets processed under different conditions at protein region of ca. 1718–1481 cm^-1^, the results were showed in Fig 4. In Fig 4(a), the unpelleted co-product was compared with all pelleted products in protein spectral region. It was observed in Fig 4(a) that no separate class can be distinguished in CLA, indicating the unprocessed mash was not completely different from pellets. This was supported by PCA in Fig 4(a), they cannot form two separate ellipses because of overlap of ellipses. More specifically, comparisons between the unprocessed mash and pellets produced under different conditions using CLA and PCA are showed in Fig 4(b) and 4(c). It was observed in Fig 4(b) that the unprocessed mash was similar to the samples conditioned at 70°C in structural make-up within protein spectral region, because no separate class and ellipse was found in CLA and PCA. Similar results were observed when unprocessed mash was compared with the samples conditioned at 80°C and 90°C, and the samples conditioned for 30s and 60s [Fig 4(b) and 4(c)], in which no distinct class can be distinguished in CLA and no grouped ellipses in PCA can be separated because of overlapped ellipses. Hence, it was summarized above that there were similar relationships between the unprocessed mash and pellets produced under different conditions with respect to protein molecular structure spectral profiles.

### Correlations between protein molecular spectral profiles and nutritive values of pelleted co-product in dairy cows

Table 1 presents the result of correlation between protein molecular spectral profiles and protein nutritive profiles, protein subfractions and energy profiles in pellet processed co-product. It was observed that CP content of the co-product was positively correlated amide II area (R = 0.77, *P* < 0.05), while negatively correlated with ratio of amide I area to amide II area (R = -0.92, *P* < 0.01), indicating that a higher amide II area and a lower area ratio of amide I to amide II were associated with a higher CP content of pelleted co-product in our study. The soluble CP content of canola meal was positively correlated with amide II height (R = -0.82, *P* < 0.05), but positively correlated with ratio of amide I to made II height (R = 0.89, *P* < 0.01). Hence, a higher amide II height or a lower ratio of amide I to amide II height was related to a lower soluble CP content. In protein subfractions, PA2 was negatively correlated with amide II height (R = -0.82, *P* < 0.05), but positively correlated with ratio of amide I to amide II height (R = 0.89, *P* < 0.01). In contrast to PA2, PB1 was positively correlated amide II height (R = 0.85, *P <* 0.05) but negatively correlated with ratio of amide I to amide II height (R = -0.94, *P* < 0.01). Additionally, PB1 was also positively correlated with (R = 0.80, *P* < 0.05) but negatively correlated with ratio of amide I to amide II area (R = -0.92, *P* < 0.01). Thus, it is indicated that protein subfractions can be associated with protein molecular structure. In estimated energy profiles, only tdCP was positively correlated with amide II area (R = 0.77, *P* < 0.05), but negatively correlated with ratio of amide I area to amide II area (R = -0.94, *P* < 0.05). Amide II related spectral profiles were more correlated with nutrient profiles of pelleted co-product, indicating that amide II played a more important role in determining nutrient profiles of pelleted co-product.

**Table 1:** Correlations between protein molecular spectral profiles and protein profiles, protein subfraction and estimated energy profiles in pelleting-processed canola meal using ATR-FT/IR.

Items	Amide I height	Amide II height	Height ratio of amide I to amide II	Amide I area	Amide II area	Area ratio of amide I to amide II	α-helix height	β-sheet height	Ratio of α-helix to β-sheet
Spearman Correlation R values									
Basic protein profiles									
CP (%DM)	0.30	0.69+	-0.71	0.21	0.77*	-0.92**	0.49	0.25	0.73+
SCP (%CP)	-0.28	-0.82*	0.89**	-0.27	-0.66	0.65	-0.40	-0.20	-0.71+
NDICP (%CP)	0.23	0.37	-0.36	0.23	0.12	0.09	0.20	0.19	0.10
ADICP (%CP)	0.32	0.17	-0.06	0.24	0.12	0.06	0.36	0.34	0.00
Protein subfractions									
PA2 (%CP)	-0.28	-0.82*	0.89**	-0.27	-0.66	0.65	-0.40	-0.20	-0.71*
PB1 (%CP)	0.25	0.85*	-0.94**	0.21	0.80*	-0.92**	0.42	0.16	0.88**
PB2 (%CP)	-0.05	0.11	0.17	0.02	-0.13	0.20	-0.14	-0.09	-0.06
PC (%CP)	0.34	0.13	0.00	0.28	0.06	0.17	0.35	0.37	-0.09
TP (%CP)	-0.34	-0.12	-0.01	-0.23	-0.06	-0.17	-0.35	-0.37	0.10
PA2_TP (%CP)	-0.27	-0.81*	0.90**	-0.25	-0.66	0.66	-0.38	-0.17	-0.72+
PB1_TP (%CP)	0.33	0.88**	-0.94**	0.28	0.83*	-0.89**	0.51	0.25	0.87*
PB2_TP (%CP)	-0.03	0.11	-0.17	0.04	-0.13	0.27	-0.12	-0.07	-0.07
Estimated energy profiles									
tdCP (%DM)	0.26	0.68	-0.72	0.18	0.77*	-0.94*	0.44	0.20	0.75
TDN_1x_, %DM	0.56	0.03	0.27	0.55	0.15	0.25	0.53	0.64	-0.32
TDN_3x_, %DM	0.55	0.02	0.27	0.54	0.13	0.27	0.51	0.63	-0.34
DE_1x_, Mcal/kg,	0.52	0.12	0.12	0.50	0.28	0.03	0.54	0.59	-0.16
DE_p3x_, Mcal/kg	0.34	0.53	-0.49	0.32	0.64	-0.62	0.45	0.35	0.28
ME_p3x_, Mcal/kg	0.45	0.44	-0.33	0.40	0.57	-0.46	0.58	0.47	0.33
NE_Lp3x_, Mcal/kg	0.44	0.61	-0.55	0.33	0.58	-0.52	0.59	0.40	0.59
ME, Mcal/kg,	-0.53	-0.46	0.31	-0.61	-0.41	0.01	-0.47	-0.60	0.27
NE_m_, Mcal/kg,	0.49	0.50	-0.37	0.38	0.56	-0.47	0.60	0.48	0.37
NE_g_, Mcal/kg,	0.38	0.12	0.04	0.31	0.22	-0.04	0.48	0.41	0.24

Notes: R: correlation coefficient calculated using spearman method; CP: crude protein; ADICP: acid detergent insoluble crude protein; NDICP: neutral detergent insoluble crude protein; SCP: soluble crude protein; PA2: rapidly degradable true protein; PB1: moderate degradable true protein. PB2: slowly degradable true protein; PC: undegradable protein; TP: true protein; PA2_TP: PA2 presents in %TP basis; PB1: PB1 presents in %TP basis; PB2_TP: PB2 presents in %TP basis; tdCP: total digestible crude protein; TDN1x: total digestible nutrients; TDN_3x_: total digestible nutrients at 3x maintenance; DE_1x_: digestible energy; DEp3x: digestible energy at a production level (3x maintenance); ME_p3x_: metabolizable energy at a production level (3x maintenance); NE_Lp3x_: Net energy at a production level (3x maintenance); ME: metabolizable energy; NE_m_: net energy for maintenance; NE_g_: net energy for gain.

“^+^”: *P* < 0.10;

“*”: *P* < 0.05

“**”: *P* < 0.01;

“***”: *P* < 0.001.

### Multi-regression to detect the most important variables among protein spectral profiles in prediction of protein and energy values in dairy cattle

Table 2 shows the results of multi-regression with spectral variables selection. The model used in multi-regression was: Y = amide I height (AIH) + amide II area (AIIA) + amide I area (AIA) + amide II height (AIIH) + ratio of amide I to amide II area (Ratio1) + ratio of amide I to amide II height (Ratio3) + α-helix (HH) +β-sheet (SH) + ratio of α-helix to β-sheet (ratio2) ….+ error. This analysis was to select most important protein structural variables in determining protein nutrient supply to dairy cows. For protein nutritional profiles, the ratio of amide I to amide II area was a better predictor for CP, taking 84% of total variance. The Ratio3 (ratio of amide I to amide II height) was the selected predictor for SCP, and 80% of total variance was taken by it. In protein subfractions, the ratio3 was the better predictor for PA2, PA2_TP and PB1_TP, and took 80, 81 and 90% of total variance, respectively. In energy estimation, the ratio1 was the only parameter left in the model for tdCP and accounted 89% of total variance.

**Table 2:** Multi-regression analysis using ATR-FT/IR with tested regression model to detect the most important variables among protein spectral profiles in predicting nutrients profiles of canola meal.

Variables in the model with *P* < 0.05	Variables in the model with *P* < 0.05	Prediction Equation Y = a + b1 × x1 + b2 × x2…..	R^2^ values	RSD	P values
Protein profiles					
CP (%DM)	Ratio1stayed in the model	CP = 44.66–1.96 × ratio1	0.84	0.12	<0.01
SCP (%CP)	Ratio3 stayed in the model	SCP = 8.61 + 8.20 × ratio3	0.80	0.43	<0.01
NDICP (%CP)	No variable met the 0.0500 significance level for entry into the model.				
ADICP (%CP)	No variable met the 0.0500 significance level for entry into the model.				
Protein subfractions					
PA2 (%CP)	Ratio3 stayed in the model	PA2 = 8.61 + 8.20 × ratio3	0.80	0.43	<0.01
PB2 (%CP)	No variable met the 0.0500 significance level for entry into the model.				
PC (%CP)	No variable met the 0.0500 significance level for entry into the model.				
TP (%CP)	No variable met the 0.0500 significance level for entry into the model.				
PA2_TP (%TP)	ratio3 stayed in the model	PA2_TP = 8.96+8.62 × ratio3	0.81	0.45	<0.01
PB1_TP (%TP)	ratio3 stayed in the model	PB1_TP = 77.51–7.92 × ratio3	0.90	0.28	<0.01
PB2_TP (%TP)	No variable met the 0.0500 significance level for entry into the model.				
Estimated energy profiles					
tdCP (%DM)	ratio1 stayed in the model	tdCP = 43.98–1.97 × ratio1	0.89	0.10	<0.01
TDN_1x_ (%DM)	No variable met the 0.0500 significance level for entry into the model.				
TDN_3x_ (%DM)	No variable met the 0.0500 significance level for entry into the model.				
DE_1x_, Mcal/kg, dairy	No variable met the 0.0500 significance level for entry into the model.				
ME_p3x_, Mcal/kg, dairy	No variable met the 0.0500 significance level for entry into the model.				
NE_lp3x_, Mcal/kg, dairy	No variable met the 0.0500 significance level for entry into the model.				

Notes: RSD: Residual standard deviation; CP: crude protein; ADICP: acid detergent insoluble crude protein; NDICP: neutral detergent insoluble crude protein; SCP: soluble crude protein; PA2: rapidly degradable true protein; PB1: moderate degradable true protein. PB2: slowly degradable true protein; PC: undegradable protein; TP: true protein; PA2_TP: PA2 presents in %TP basis; PB1: PB1 presents in %TP basis; PB2_TP: PB2 presents in %TP basis; tdCP: total digestible crude protein; TDN_1x_: total digestible nutrients; TDN_3x_: total digestible nutrients at 3x maintenance; DE1x: digestible energy; DE_p3x_: digestible energy at a production level (3x maintenance); ME_p3x_: metabolizable energy at a production level (3x maintenance); NEL_p3x_: Net energy at a production level (3x maintenance); ME: metabolizable energy; NE_m_: net energy for maintenance; NE_g_: net energy for gain. Ratio1: ratio of amide I to amide II area; ratio3: ratio of amide I to amide II height.

In summary, the molecular structure of the co-product (canola meal) associated with functional groups cannot be assessed by using conventional chemical analysis methods. With application of ATR-FT/IR and use of both univariate and multivariate molecular analyses, it is possible to detect inherent structure changes that were caused by pelleting under different conditions. The ATR-FTIR required little amount of samples, and samples ground for chemical analysis can be directly used in ATR-FT/IR. The analysis using ATR-FT/IR was rapid and non-destructive. In addition, the analysis of spectral data using multivariate analysis was time saving and required no prior knowledge of spectroscopy.

Combined results of univariate and multivariate molecular analyses showed that pelleting process under different conditions was able to alter inherent structures of functional groups in the co-product. The results of univariate molecular analysis indicated that absorption intensities of protein related chemical functional groups and relative ratios between different absorption bands in co-product were affected by the pelleting process in this study.

The results of multivariate analysis indicated that inherent structural characteristics of all functional groups were not fully distinguished. Hence, it was demonstrated that pelleting process under different conditions in this study was able to cause partial changes in the co-product in spectral characteristics of functional groups, but these changes were not sufficient to make the entire spectral region of functional groups become fully distinguishable. This was because only partial spectral information in a specific region was assessed by univariate molecular analysis, while multivariate analysis considered the entire spectral information within a specific spectral region.

Protein molecular spectral profiles were correlated with protein subfractions of pelleted co-product in the current study. The result of multi-regression demonstrates that protein molecular structure plays important roles in determining protein nutritive profiles. Multi-regression study showed that it is possible to predict protein profiles in co-product based on protein molecular structure spectral profiles. This novel approach provides us a new way to evaluate feed protein value based on molecular structure of protein which can be rapidly determined by vibrational molecular spectroscopy. Are there any benefits to having this new way? Should people change what they are doing?

## Abbreviations

ADF: Acid detergent fibre
ADICP: Acid detergent insoluble crude protein
ADL: Acid detergent lignin
AIA: Amide I area
AIH: Amide I height
AIIA: Amide II area
AIIH: Amide II height
ATR: Attenuated total reflectance
CA: Cellulosic compounds area in spectral profiles
CA1: Volatile fatty acids
CA2: Lactic acids with degradation rate of 7%/h
CA3: Organic acids with degradation rate of 5%/h
CA4: Simple sugars with degradation rate of 40–60%/h
CB1: Starch with degradation rate of 20–40%/h
CB2: Soluble fibre with degradation rate of 20–40%/h
CB3: Available NDF with degradation rate of 4–9%/h
CC: Unavailable cell wall
CHO: Carbohydrate
CLA: Hierarchical cluster analysis
CNCPS: Cornell net carbohydrate and protein system
CP: Crude protein
DE: Digestible energy
DE1x: Digestible energy at 1x maintenance
DM: Dry matter
DOM: Digestible organic matter
EE: Ether extract
FMV: Feed milk value
FTIR: Fourier transform infrared microspectroscopy
ME: Metabolizable energy
MEp: Metabolizable energy at different a production levels
MP: Metabolizable protein
N: Nitrogen
NDF: Neutral detergent fibre
NDICP: Neutral detergent insoluble crude protein
NE: Net energy
NEg: Net energy for growth
NELp: Net energy for lactation at production level
NEm: Net energy for maintenance
NFC: Non-fibrious carbohydrate
NPN: Non protein nitrogen
PA1: Ammonia
PA2: Non-ammonia soluble protein
PB1: Moderately degraded protein with degradation rate of 3–20%/h
PB2: Slowly degraded protein with degradation rate of 4–9%/h
PC: Unavailable protein
PCA: Principal component analysis
PDI: Pellet durability index
R: Correlation coefficient using Spearman correlation
Ratio1: Ratio of amide I to amide II area
Ratio2: Ratio of α-helix to β-sheet
Ratio3: Ratio of amide I to amide II height
RCBD: randomized complete block design
SCP: Soluble crude protein
tdCP: Truly digestible CP
tdFA: Truly digestible fatty acid
TDN: Total digestible nutrients
TDN1x: Total digestible nutrients at 1x maintenance
TDNDF: Total digestible NDF
tdNFC: Truly digestible NFC
